# Surrogate Immunohistochemical Markers of Proliferation and Embryonic Stem Cells in Distinguishing Ameloblastoma from Ameloblastic Carcinoma

**DOI:** 10.1007/s12105-024-01704-8

**Published:** 2024-10-04

**Authors:** Liam Robinson, Chané Smit, Marlene B. van Heerden, Haroon Moolla, Amir H. Afrogheh, Johan F. Opperman, Melvin A. Ambele, Willie F. P. van Heerden

**Affiliations:** 1 Department of Oral and Maxillofacial Pathology, Faculty of Health Sciences, University of Pretoria, Pretoria Oral Health Care Centre, Office 6-11, Corner of Steve Biko and Dr Savage Roads, Pretoria, 0084 South Africa; 2 Centre for Infectious Disease Epidemiology and Research, Faculty of Health Sciences, University of Cape Town, Cape Town, South Africa; 3 Department of Oral and Maxillofacial Pathology, Faculty of Dentistry, University of the Western Cape, Cape Town, South Africa; 4 Division of Anatomical Pathology, Faculty of Health Sciences, Stellenbosch University, Cape Town, South Africa; 5 Institute for Cellular and Molecular Medicine, Extramural Unit for Stem Cell Research and Therapy, Faculty of Health Sciences, South African Medical Research Council, University of Pretoria, Pretoria, South Africa; 6 PathCare Vermaak Histopathology Laboratory, Pretoria, South Africa

**Keywords:** Odontogenic neoplasms, Ameloblastoma, Ameloblastic carcinoma, Immunohistochemistry, Proliferation indices, Stem cells

## Abstract

**Purpose:**

The current study aimed to investigate the use of surrogate immunohistochemical (IHC) markers of proliferation and stem cells to distinguish ameloblastoma (AB) from ameloblastic carcinoma (AC).

**Methods:**

The study assessed a total of 29 ACs, 6 ABs that transformed into ACs, and a control cohort of 20 ABs. The demographics and clinicopathologic details of the included cases of AC were recorded. The Ki-67 proliferation index was scored through automated methods with the QuPath open-source software platform. For SOX2, OCT4 and Glypican-3 IHC, each case was scored using a proportion of positivity score combined with an intensity score to produce a total score.

**Results:**

All cases of AC showed a relatively high median proliferation index of 41.7%, with statistically significant higher scores compared to ABs. ABs that transformed into ACs had similar median proliferation scores to the control cohort of ABs. Most cases of AC showed some degree of SOX2 expression, with 58.6% showing high expression. OCT4 expression was not seen in any case of AC. GPC-3 expression in ACs was limited, with high expression in 17.2% of ACs. Primary ACs showed higher median proliferation scores and degrees of SOX2 and GPC-3 expression than secondary cases. Regarding SOX2, OCT4 and GPC-3 IHC expression, no statistically significant differences existed between the cohort of ABs and ACs.

**Conclusion:**

Ki-67 IHC as a proliferation marker, particularly when assessed via automated methods, was helpful in distinguishing AC from AB cases. In contrast to other studies, surrogate IHC markers of embryonic stem cells, SOX2, OCT4 and GPC-3, were unreliable in distinguishing the two entities.

## Introduction

Odontogenic tumours encompass a group of continuously evolving entities derived from remnants of the tooth germ [1, 2]. Most odontogenic tumours are considered neoplastic and subdivided into benign and malignant entities [3]. Of these odontogenic neoplasms, the majority fall into the benign category, whereas malignant odontogenic entities are significantly rarer [1, 2].

Definitions and classifications of malignant odontogenic neoplasms have changed over the years, emanating in the latest 5th Edition of the WHO Classification of Head and Neck Tumors released in 2022 [3]. This Edition included ameloblastic carcinoma (AC), primary intraosseous carcinoma (not otherwise specified), sclerosing odontogenic carcinoma, clear cell odontogenic carcinoma (CCOC), ghost cell odontogenic carcinoma, odontogenic sarcomas, and odontogenic carcinosarcoma within the category of malignant odontogenic tumours. AC is the most common odontogenic malignancy, constituting approximately 30% of all cases in this category. The 5th Edition of the WHO classification vaguely defines AC as a primary odontogenic carcinoma histologically resembling ameloblastoma (AB) [3]. ACs are further subdivided into primary cases that arise de novo, and secondary cases arising in an untreated or recurrent AB [1, 3–6].

The rarity of ACs, paired with their poorly defined diagnostic threshold, can make their subsequent diagnosis particularly challenging [1, 2]. Authors have proposed various ancillary studies, including immunohistochemistry (IHC), to assist in diagnosing difficult cases [7]. Scoring the tumour's proliferation index via Ki-67 IHC is one of the earliest methods utilised to distinguish benign versus malignant odontogenic tumours [7–9]. Ki-67 is a protein expressed by proliferating cells in the various stages of the cell cycle, except for the resting phase [9, 10]. Additionally, several more recent studies have investigated surrogate IHC markers of embryonic stem cells, in particular, SOX2, but also OCT4 and Glypican-3, and their role in the oncogenesis, diagnosis and treatment of cases of ameloblastic carcinoma and other aggressive odontogenic entities [11–14].

The SRY-related high-mobility-group box (SOX) family of transcription factors consists of 20 protein members [11]. More specifically, the SOX2 gene functions in humans as an established transcription factor that modulates embryonic stem cell self-renewal and differentiation [15–17]. The function of these essential biological processes relies on the interaction of SOX2 with several other transcription factors, including OCT4 [15]. Of significance, SOX2 is also involved in many functions related to carcinogenesis, including promoting tumour cell proliferation, the ability to repress apoptosis, accelerating cell invasion and migration, regulating self-renewal of tumour stem cells, and metastatic potential [12, 17–19]. SOX2 expression has been linked to staging, relapse, therapy resistance, and overall prognosis in several human cancers, including lung, ovarian, urothelial, breast, pancreatic, colorectal, oesophageal, nasopharyngeal and even oral squamous cell carcinoma [12, 17, 18, 20–26]. Studies have also shown that SOX2 may be regulated at a transcriptional level via epigenetics, leading to SOX2 silencing in some human cancers, correlating to more aggressive biological behaviour and poorer overall prognosis [18, 27–29].

Octamer-binding transcription factor 4 (OCT4) is a member of the family of POU (Pit-Oct-Unc) domain transcription factors [30–34], and functions in regulating the expression of target genes by binding to either promoter or enhancer regions on the octamer motif [34]. OCT4 is found in undifferentiated pluripotent cells, promoting the expression of stem cell-specific genes in combination with several other transcription factors. It is also involved in chromatin regulation, cell cycle control, apoptosis, and DNA repair [34]. It is absent in most somatic cells but has been linked to oncogenesis, with overexpression in several cancers, including ovary, lung, liver, breast, colorectal, and brain [30, 34–36].

Glypicans are a family of heparan sulfate proteoglycans comprising six members, termed GPC 1–6 [13, 37–40] and, depending on their biological stimulus, either stimulate or inhibit cell signalling activity [37–40]. More specifically, the GPC-3 gene encodes a 70-kDa surface protein, which shows high levels of expression in embryonic tissue [40, 41]. Diagnostically, GPC-3 immunohistochemistry is a marker of hepatocellular carcinoma and, more recently, a potential target for antineoplastic treatment [42–44].

Unfortunately, specific research pertaining to malignant odontogenic tumours is limited and often conflicting; therefore, additional, more robust studies are required. The current study aims to investigate the use of surrogate IHC markers of proliferation and stem cells in distinguishing AB from AC. The findings of this study will hopefully advance the current understanding of the aetiopathogenesis of these odontogenic carcinomas and ultimately aid in improving their diagnosis.

## Materials and Methods

### Case Selection

The histopathologic database of the Department of Oral and Maxillofacial Pathology at the University of Pretoria was searched for cases diagnosed as ameloblastic carcinoma between 2002 and 2022 (20-year period). The principal investigator (LR) and an experienced Oral and Maxillofacial Pathologist (WvH) reviewed all cases to confirm the diagnosis of ameloblastic carcinoma according to the 2022 WHO Classification diagnostic criteria. These essential diagnostic criteria include a histopathologic resemblance to AB and evidence of cytologic atypia. Only intraosseous jaw tumours were included in the current study. Although only considered a desirable diagnostic feature, both the presence and character of tumour necrosis was recorded in all cases [3]. Furthermore, the presence or absence of perineural invasion was recorded, and mitotic figures were counted per 2mm^2^ (10 high power fields with a field diameter of 0.55 mm).

The original haematoxylin and eosin (H&E)-stained slides, IHC stains, and the formalin-fixed paraffin-embedded (FFPE) tissue blocks were retrieved from the Departmental archives. The best representative FFPE tissue block was selected to perform additional IHC studies. These FFPE tissue blocks were stored at temperatures maintained in the range of 17–22 °C and protected from direct light to maintain tissue integrity. Tissue sections that were decalcified or contained osseous material were not used for further ancillary testing.

Once a case met the essential criteria for a diagnosis of AC, the histopathologic database was re-assessed to subclassify the case as either a primary or a secondary AC (arising ex ameloblastoma). Records of a previous diagnosis of AB were required for secondary cases of AC. If available, the previous diagnosis of AB was re-assessed and confirmed.

A control cohort of 20 conventional ABs was also selected from the same histopathologic database of the Department within the same study period. These cases encompassed the histopathologic spectrum of conventional ABs and included both mandibular and maxillary cases. All cases were re-assessed by the principal investigator and an experienced Oral and Maxillofacial Pathologist (WvH) to confirm the diagnosis as stipulated by the 2022 WHO Classification [3].

The study was conducted following approval by the University of Pretoria, Faculty of Health Sciences Research Ethics Committee (Reference number: 228/2023). All procedures followed were in accordance with the ethical standards of the responsible committee on human experimentation (institutional and national) and with the Helsinki Declaration of 1975, as revised in 2008.

### Immunohistochemical Technique

Immunohistochemical staining (Ki-67, SOX2, OCT4 and GPC-3) was performed on the following:

- Ameloblastic carcinomas (primary and secondary);
- Ameloblastomas that transformed into ameloblastic carcinomas;
- A cohort of conventional ameloblastomas.

Immunohistochemical staining was performed according to the manufacturer's instructions (Table 1) on freshly cut 4 µm sections from the representative FFPE tissue block. Staining was performed on a Benchmark XT automated System (Ventana Medical Systems, Inc. Tuscan, Arizona USA Roche). Mild antigen retrieval was performed using CC1 cell conditioning buffer followed by incubation with the primary antibody for 25 min at 37 °C. The antigen–antibody binding sites were detected using the Ventana ultraView DAB kit. The sections were then counterstained with haematoxylin and mounted with DPX permanent mounting media.

**Table 1:** Characteristics of immunohistochemical antibodies used in this study

Antibody	Supplier	Dilution	Clone	Antibody Incubation Time	Positive Control Sample	Staining Pattern
Ki-67	Dako, USA	RTU	MIB1	30 min	Appendix	Nuclear
SOX2	Cell Signalling Technologies, USA	1:400	D6D9	25 min	Cervical squamous cell carcinoma	Nuclear
OCT4	Cell Marque, Sigma-Aldrich, USA	RTU	MRQ-10	25 min	Seminoma	Nuclear
Glypican-3	Cell Marque, Sigma-Aldrich, USA	RTU	IG12	25 min	Hepatocellular carcinoma	Cytoplasmic and membranous

*RTU* Ready to use

### Immunohistochemical Analysis

**Fig. 1:**
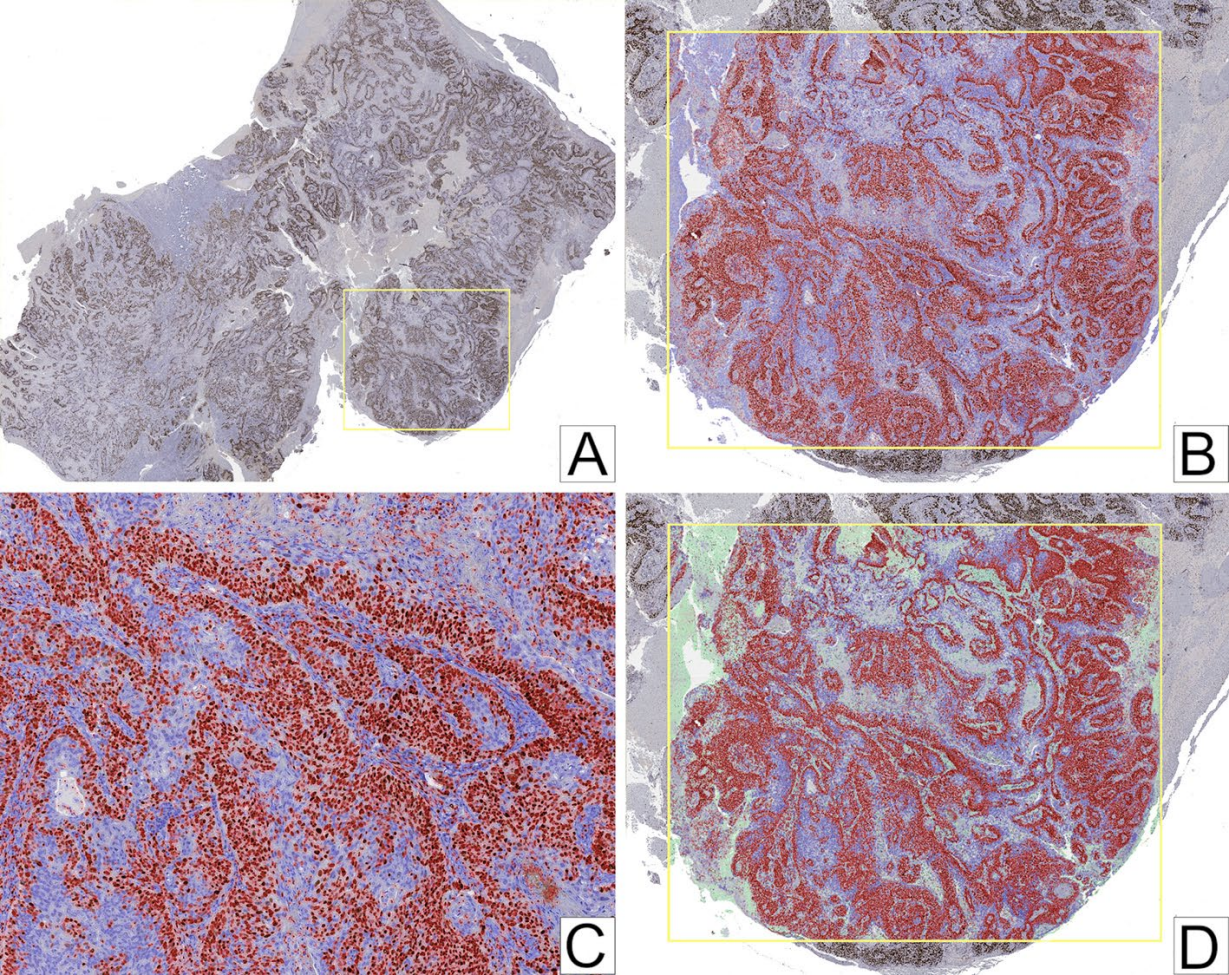
QuPath methodology for whole slide image analysis. **A** Selection of a rectangular region of interest in a hotspot area. **B** Positive nuclear cell detection with positive cells marked red and negative cells marked purple. **C** Manual verification of positive and negative nuclear cell detection. **D** Training object classifier to distinguish between stroma (green) and tumour (with positive [red] and negative [purple] cells) to determine the percentage of positively stained tumour cells alone

For Ki-67 IHC analysis, the glass slides were digitally scanned using the Aperio CS2 slide scanner (Leica Biosystems). The SVS image files were imported into QuPath open-source software platform, version 0.5.1, for whole slide image analysis. Upon importation, the Brightfield (H-DAB) stain option was selected. A rectangular region of interest (ROI), corresponding to an area measuring 20 × 10^6^ μm^2^, was selected in a hotspot region. Positive nuclear cell detection was run within the ROI, after which it was manually verified. If the program over- or under-estimated the positive cells, the cell intensity classifier threshold was adjusted until calibration was achieved. Next, the object classifier was trained by manually selecting and labelling areas of stroma and tumour using the polygon tool. After the selection process, the object classifier was trained to distinguish between stroma and tumour. The accuracy of stroma and tumour distinction was then manually verified. If areas were incorrectly classified, more areas were manually selected and labelled accordingly, and the program trained again until accuracy was achieved. After this, positive nuclear cell detection was run once again, and the percentage of positively stained tumour cells within the hotspot ROI was recorded (Fig. 1). Overall, a mean number of 73,371 nuclei was counted during the analysis.

For SOX2, OCT4 and Glypican-3 IHC, five fields were selected for analysis. The estimated proportion of positivity (PP) was calculated as follows: score 0 (0%), score 1 (> 0– < 25%), score 2 (25–50%), score 3 (51–75%), and score 4 (> 75%). An intensity score (IS) was also calculated as follows: score 0 (no expression), score 1 (weak), score 2 (moderate), and score 3 (strong). The total score (TS) was then calculated, where TS = PP + IS. Therefore, each case had a TS that ranged from 0 to 7 points, which was then further categorised into three groups: no expression, low expression (< 4 points) and high expression (4–7 points) [14].

### Statistical Analysis

This study included three sets of comparisons: primary ACs compared to secondary ACs, conventional ABs that transformed into ACs compared to those that did not, and conventional ABs compared to ACs. In all comparisons, Ki-67, SOX2, OCT4, and GPC-3 IHC were assessed. Differences in necrosis type, mitotic count, and perineural invasion were also evaluated to compare primary and secondary ACs.

Appropriate descriptive statistics were performed for all variables, including means and standard deviations (or medians and interquartile ranges for non-normally distributed data) for numerical variables, and frequencies and percentages for categorical variables. For the numerical variables, Ki-67 and mitotic count, unpaired t-tests or Mann–Whitney U tests were used depending on the normality of their distributions (assessed using the Shapiro–Wilk test). The categorical variables SOX2, OCT4, GPC-3, and necrosis type were compared using Fisher's exact test because the contingency tables contained cells with counts less than 5. The binary variable perineural invasion was compared using the two-sample Z test of proportions.

All statistical tests were two-sided, and p-values less than 0.05 were considered significant. Data analysis was conducted using Stata (version 17.0, StataCorp, Texas, USA).

## Results

During the 20-year study period, a total of 29 cases of ACs were recorded, with 17 cases diagnosed as primary ACs, and 12 diagnosed as secondary ACs arising from a preexisting AB. Six ABs that transformed to AC had available FFPE tissue blocks for additional ancillary tests.

Table 2 summarises the main demographic and clinical features of the included cases of AC and compares the results to a recent systematic review of ACs [45]. The mean age of patients diagnosed with AC in the current study was younger than that of the systematic review. The male-to-female ratio in the current sample showed a male predominance. The mean duration of the tumour, as reported by the patient, was higher than that of the systematic review. Most cases involved the mandible, with a predilection for the posterior region, although several cases extended to involve both the anterior and posterior regions. Maxillary cases were also prevalent, comprising 38% of the cohort. Intrabony swellings were more frequent than the cases reported in the literature, with reported associated pain being less frequent. Radiologically, cases presented with higher rates of poorly-demarcated borders, with nearly all cases presenting as radiolucent lesions. Multilocular lesions predominated, with higher percentages of cortical destruction and associated tooth displacement and root resorption compared to the review.

**Table 2:** Summarised demographic data and clinical features of ameloblastic carcinomas

	Current study		Systematic review		Total	
Demographic/clinical features	n = 29	%	n = 285	%	n = 314	%
Age (years)—mean, range	43	4.0–76.0	46.1	2.0–93.0	45.8	2.0–93.0
Sex (M:F)	20:9	2:1	95:189	1:2	115:198	1:1.7
Clinical duration of the lesion (months)—mean, range^a^	40	1.0–168.0	28.3	0.0–372.0	29.7	0.0–372.0
**Site**						
Mandible	18	62.1%	202	71.1%	220	70.1%
Maxilla	11	37.9%	82	28.9%	93	29.6%
Anterior^b^	6	23.1%	26	11.9%	32	13.1%
Posterior^b^	11	42.3%	140	64.2%	151	61.9%
Both^b^	8	30.8%	42	19.3%	50	20.5%
**Clinical signs and symptoms**^c^						
Swelling	22	95.7%	107	49.5%	129	54.0%
Painful	3	13.0%	88	40.7%	91	38.1%
Ulceration	3	13.0%	15	6.9%	18	7.5%
Tooth mobility	5	21.7%	5	2.3%	10	4.2%
**Radiologic features**^d^						
**Borders**						
Well-demarcated	7	31.8%	72	47.4%	79	45.4%
Poorly-demarcated	15	68.2%	80	52.6%	95	54.6%
**Radiodensity**						
Radiolucent	21	95.5%	153	96.2%	174	96.1%
Internal calcifications	1	4.5%	2	1.3%	3	1.7%
Mixed (radiolucent–radiopaque)	0	0.0%	4	2.5%	4	2.2%
**Locularity**						
Unilocular	6	27.3%	89	58.6%	95	54.6%
Multilocular	16	72.7%	63	41.4%	79	45.4%
**Bone effects**						
Cortical destruction	20	90.9%	97	78.9%	117	80.7%
**Tooth effects**						
Tooth displacement	6	27.3%	11	14.5%	17	17.3%
Root resorption	9	40.9%	25	32.9%	34	34.7%

^a^Duration was not reported in 12 cases in the current sample. ^b^2 mandibular and 1 maxillary case did not specify the subsite in the current sample. ^c^Signs/symptoms not reported in 6 cases in the current sample. ^d^7 cases did not have radiograph/radiologic description available in the current sample

**Fig. 2:**
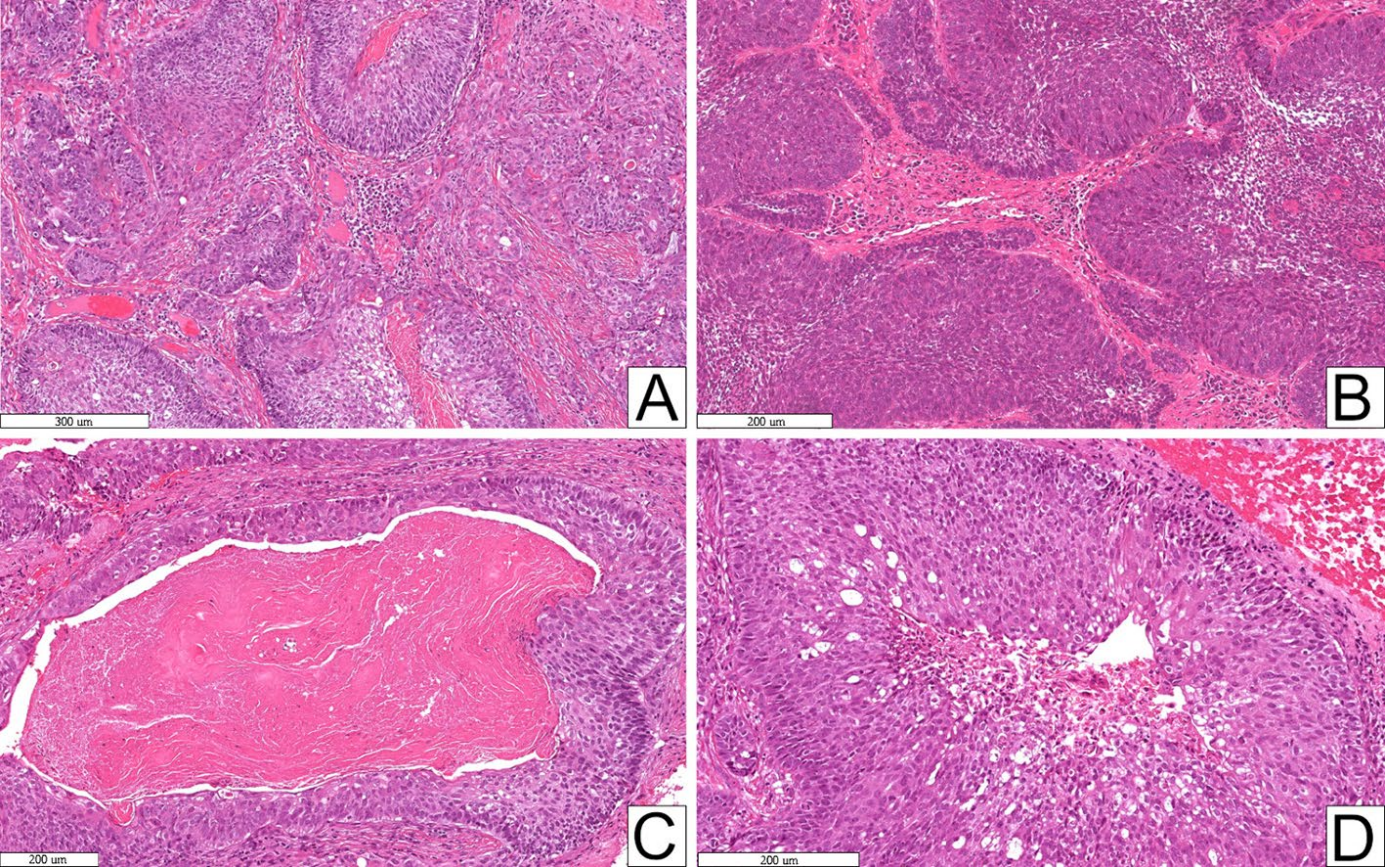
Histopathologic features of ameloblastic carcinomas. **A** Essential diagnostic criteria, including resemblance to ameloblastoma and cytologic atypia (original magnification × 40). **B** Evidence of extensive basal cell crowding (original magnification × 40). Desirable diagnostic criteria of tumour necrosis—presenting as either **C** Comedo-type necrosis (original magnification × 100) or **D** Focal/punctate necrosis (original magnification × 100)

**Fig. 3:**
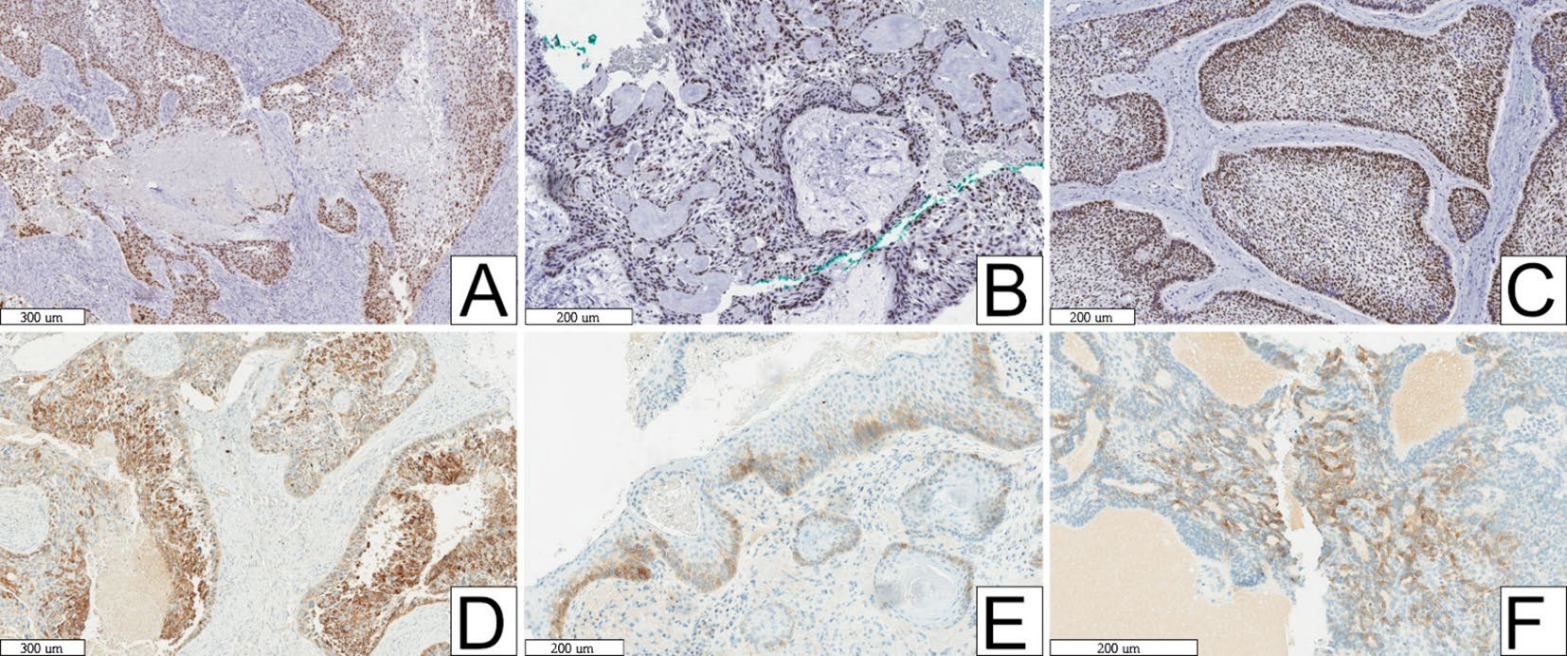
Immunohistochemical markers of stem cells. Representative high SOX2 IHC expression in **A** Ameloblastic carcinoma (original magnification × 100), **B** Ameloblastoma that transformed into ameloblastic carcinoma (original magnification × 100), and **C** Ameloblastoma (original magnification × 100). Representative high GPC-3 IHC expression in **D** Ameloblastic carcinoma (original magnification × 100), **E** Ameloblastoma that transformed into ameloblastic carcinoma (original magnification × 200), and **F** Ameloblastoma (original magnification × 100)

The histopathologic features and IHC results of included cases of primary and secondary ACs are summarised in Table 3. All cases diagnosed as AC met the essential diagnostic criteria according to the 5th Edition of the WHO Classification [3] (Fig. 2A, B). All cases of AC showed some degree of necrosis, either comedo-type necrosis or areas of focal/punctate necrosis (Fig. 2C, D). Primary ACs showed statistically significant higher mean mitotic counts than cases of secondary AC. Perineural invasion was also higher in primary ACs. Regarding Ki-67 IHC, all ACs showed a relatively high median proliferation index of 41.7%, with an interquartile range of 29.1–59.9%. Primary ACs showed an overall higher median score. Most cases of AC showed some degree of SOX2 expression, with 58.6% showing high expression (Fig. 3). OCT4 expression was not seen in any case of AC. Most cases of AC, whether primary or secondary, showed no expression of GPC-3. Primary ACs showed higher degrees of SOX2 and GPC-3 expression compared to secondary cases (Fig. 3).

**Table 3:** Histopathologic features and immunohistochemical results of ameloblastic carcinomas

Histopathologic features	Secondary ACs		Primary ACs		p-value	Total ACs	
	n = 12	%	n = 17	%		n = 29	%
Necrosis					0.876		
*Focal/punctate*	6	50.0%	8	47.1%		14	48.3%
*Comedo*	6	50.0%	9	52.9%		15	51.7%
Mitosis [mean, (standard deviation), (range)]	8.3 (3.2)	(5–16)	11.4 (4.3)	(6–19)	0.042*	10.1(4.2)	(5–19)
Perineural invasion	5	41.7%	10	58.8%	0.363	15	51.7%
**Immunohistochemistry**							
**Ki-67** (median, interquartile range)	39.9%	(22.2–45.7%)	46.3	(32.6–62.9%)	0.132	41.7%	(29.1–59.9%)
**SOX2**							
*No expression*	1	8.3%	3	17.6%	0.093	4	13.8%
*Low expression*	6	50.0%	2	11.8%		8	27.6%
*High expression*	5	41.7%	12	70.6%		17	58.6%
**OCT4**							
*No expression*	12	100%	17	100%	N/A	29	100%
**GPC-3**							
*No expression*	9	75.0%	11	64.7%	0.725	20	69.0%
*Low expression*	2	16.7%	2	11.8%		4	13.8%
*High expression*	1	8.3%	4	23.5%		5	17.2%

^*^Statistically significant

The cohort of 20 ABs included for comparison, and the six ABs that transformed into ACs both met the essential diagnostic criteria for conventional ABs according to the 5th Edition of the WHO Classification [3]. The IHC results comparing ABs to ABs that transformed are summarised in Table 4. ABs that transformed had similar median Ki-67 proliferation rates to the control cohort of ABs. Regarding SOX2, OCT4 and GPC-3 IHC expression, no statistically significant differences existed between these two cohorts (Fig. 3).

**Table 4:** Immunohistochemical results of conventional ameloblastomas

Immunohistochemistry	Ameloblastomas		Transformed ABs		p-value	Total ABs	
	n = 20	%	n = 6	%		n = 26	%
**Ki-67** (median, interquartile range)	9%	(2–12.5%)	10%	(5–20%)	0.339	9%	(2–15%)
**SOX2**							
*No expression*	1	5.0%	1	16.7%	0.627	2	7.7%
*Low expression*	6	30.0%	2	33.3%		8	30.8%
*High expression*	13	65.0%	3	50.0%		16	61.5%
**OCT4**							
*No expression*	19	95.0%	6	100.0%	> 0.999	25	96.2%
*Low expression*	0	0.0%	0	0.0%		0	0.0%
*High expression*	1	5.0%	0	0.0%		1	3.8%
**GPC-3**							
*No expression*	17	85.0%	5	83.3%	> 0.999	22	84.6%
*Low expression*	1	5.0%	0	0.0%		1	3.8%
*High expression*	2	10.0%	1	16.7%		3	11.5%

Table 5 summarises the IHC results of conventional ABs versus ACs. ACs showed statistically significant higher median Ki-67 proliferation rates compared to ABs. Regarding SOX2, OCT4 and GPC-3 IHC expression, no statistically significant differences existed between the two cohorts (Fig. 3).

**Table 5:** Immunohistochemical results of conventional ameloblastomas vs. ameloblastic carcinomas

Immunohistochemistry	Total ABs		Total ACs		p-value
	n = 26	%	n = 29	%	
**Ki-67** (median, interquartile range)	9%	(2–15%)	41.7%	(29.1–59.9%)	< 0.001*
**SOX2**					
*No expression*	2	7.7%	4	13.8%	0.850
*Low expression*	8	30.8%	8	27.6%	
*High expression*	16	61.5%	17	58.6%	
**OCT4**					
*No expression*	25	96.2%	29	100%	0.473
*Low expression*	0	0.0%	0	0.0%	
*High expression*	1	3.8%	0	0.0%	
**GPC-3**					
*No expression*	22	84.6%	20	69.0%	0.425
*Low expression*	1	3.8%	4	13.8%	
*High expression*	3	11.5%	5	17.2%	

^*^Statistically significant

## Discussion

Malignant odontogenic tumours are rare entities, of which AC is the most common entity within this category [1, 3, 6]. ACs with evidence of frank malignant features rarely pose diagnostic challenges. Instead, difficulty exists in diagnosing cases showing intermediate histopathologic features between benign and malignant odontogenic neoplasms, likely due to poorly defined diagnostic thresholds. Akrish et al. suggested that the differential diagnosis between AB and AC should depend on integrating the histopathologic features with patient demographics and overall biological behaviour [46], a practice supported by many diagnostic histopathologists. However, in the past decade, some researchers have proposed various ancillary studies, including immunohistochemistry, to assist in diagnosing challenging cases.

In the current study, the mean age of patients with AC was 43 years, slightly lower than reported in a recent systemic review [45]. The wide age range of ACs is well documented, with a case reported in a patient as young as two years [47]. The mean clinical duration of the lesion in the current study was significantly higher than reported in the literature [45]. This is likely due to the patient cohort originating from a developing country, whereby patients present later for many reasons, including financial, travel, social, and healthcare constraints. Additionally, the mean age and clinical duration of ACs were significantly higher than ABs in the same population group [48]. This may be explained by including secondary ACs with longer overall clinical durations in the study sample. The current study had a male-to-female ratio of approximately 2:1, contradictory to the reported combined data [45]. This finding is noteworthy, as a sizeable single-centre study on ABs within the same population group found an almost equal male-to-female ratio [48]. In the current study, most cases of AC involved the posterior mandible, a finding mirrored in the reported literature. Most patients presented with intrabony swellings, with associated pain only reported in three cases. In contrast, associated ulceration and tooth mobility were frequently noted in the current cohort, likely relating to the delayed presentation and advanced stage of the disease process. The radiologic features of ACs in the current study differed from the systemic review, with more cases showing poorly-demarcated borders, higher ratios of multilocular lesions and higher frequencies of cortical destruction [45]. This may be due to the longer reported duration in the current sample leading to lesion advancement. Furthermore, when comparing the radiologic features of ACs to ABs, ACs showed a higher percentage of lesions with poorly-demarcated borders (68.2% vs. 1.9%) and evidence of cortical destruction (90.9% vs. 55.5%) [48]. Interestingly, there was a near-equal distribution of uni- and multilocular lesions in ACs in the literature compared to ABs [45, 48]. This is a significant finding, as unilocularity is often perceived as an indolent feature by clinicians.

All cases in the current study met the essential diagnostic criteria stipulated in the latest Edition of the WHO Classification [3]. Unfortunately, the rarity of ACs has resulted in limited studies, which may have contributed to vague diagnostic thresholds [1, 6]. Tumour necrosis is only considered a desirable diagnostic feature. In the current study, all cases of AC showed some degree of necrosis, whether comedo-type necrosis or areas of focal/punctate necrosis. This noteworthy finding suggests that tumour-associated necrosis, paired with the other essential criteria, may be valuable in diagnosing AC. The current study found a statistically significant higher mean mitotic count, higher incidence of perineural infiltration, higher Ki-67 values, and higher expression of SOX2 and GPC-3 in primary AC cases compared to secondary cases. This may imply that primary ACs exhibit more aggressive biological behaviour than secondary cases. Further research is required in this regard to substantiate these findings.

Ki-67 IHC gives insight into the proliferative potential of a tumour and has, therefore, been used as a diagnostic tool to differentiate different entities with similar histopathologic appearances, including AB and AC [7]. Unfortunately, the reported Ki-67 proliferation index of ACs varies considerably [7, 14], with a systematic review finding a vast range between 5 and 80% [45]. This wide percentage range may be partly due to the subjective bias in the interpretation of the stain, but it still raises questions about the utility of this IHC marker in distinguishing cases of AB from AC. Nevertheless, Yoon et al. and Niu et al. found that cases of AC had a comparably higher proliferation index than ABs [5, 49]. In the current study, a statistically significant higher Ki-67 score was seen in cases of AC compared to ABs included in the study (median of 9%). The Ki-67 score for ABs in this sample corresponded to another large AB study [50]. This emphasises the potential use of Ki-67 as a proliferation marker in distinguishing AC from AB cases. Additionally, the automated proliferation index counter used in the current study helped reduce the subjective interpretation of the IHC stain, reducing the reported range compared to the systematic review [45].

To date, there has been conflicting data in the literature regarding SOX2 expression in cases of AB and AC. A study by Juuri et al. found that SOX2 is expressed in cases of AB, regardless of the variant. The expression pattern was seen in the majority of pre-ameloblast-like cells as well as the stellate reticulum-like cells [51]. A study by Silva et al. found greater SOX2 expression in cases of odontogenic keratocyst (OKC) compared with cases of AB. They postulated that the higher expression in cases of OKC might indicate that OKC cells have significant self-renewal and proliferative properties [52]. A study by Lei et al. investigated SOX2 expression in cases of AB, atypical AB and AC. They found that strong and diffuse nuclear expression of SOX2 is a specific (86%) and sensitive (77%) marker for AC. SOX2 was essentially negative in most cases of AB [14]. The authors recommended using SOX2 in conjunction with Ki-67 in a panel to diagnose ameloblastic neoplasms. A similar study by Sobhy et al. found that SOX2 was not expressed in benign odontogenic tumours [53]. A study by Hasan et al. found SOX2 expression in 47.5% of ABs, in contrast to expression in 93% of ACs [54]. In contrast to the findings of Lei et al.[14], Sobhy et al.[53], and Hasan et al.[54], a study by Tseng et al., using the same SOX2 antibody, found nuclear SOX2 positivity in cases of AB, most prominently in the peripheral cells [12]. In the current study, SOX2 was expressed in both AB and AC cases. The expression was higher overall in cases of AB compared to ACs, demonstrating limited use of this marker in distinguishing the two entities. Additionally, SOX2 expression showed no difference in the cohort of ABs compared to ABs that ultimately transformed into ACs.

Few studies have investigated OCT4 expression in odontogenic entities [55]. A study by Banerjee et al. demonstrated OCT4 expression in dentigerous and radicular cysts and a single case of AC [32]. Monroy et al. investigated OCT4 expression in three odontogenic lesions: OKCs, adenomatoid odontogenic tumours and conventional ABs. They found nuclear and cytoplasmic expression, linking nuclear expression to so-called ‘stem-cellness’ [30]. A study by Martins Balbinot et al*.* found expression of OCT4 in the neoplastic ameloblastomatous epithelium. In contrast, a study by Bandyopadhyay et al. found no evidence of OCT4 expression in cases of OKC or AB [33]. Phattarataratip et al.[11] and Chacham et al.[56] found that apart from OKCs, other odontogenic cysts and tumours did not express OCT4. In the current study, OCT4 expression was restricted to a single case of AB, whereas no expression was seen in any of the included ACs. This supports the findings of Phattarataratip et al.[11] and Chacham et al.[56], highlighting the limited expression of OCT4 IHC in ameloblastomatous odontogenic tumours.

GPC-3 has a role in odontogenesis, negatively regulating the Hedgehog signalling pathway [57]. Utilising this concept, Mendes et al. [13] found that conventional ABs and OKCs of sporadic and syndromic origin all showed some degree of expression. The staining pattern varied with each odontogenic lesion, with conventional ABs showing expression in the peripheral columnar cells and the central cells resembling the primitive stellate reticulum [13]. A 2021 study by Hasan et al. evaluated GPC-3 expression in non-recurrent and recurrent ABs and ACs. The expression pattern was similar to the study by Mendes et al., with all cases of recurrent AB showing higher levels of expression than conventional non-recurrent cases [54]. Additionally, the study found that GPC-3 expression in AC cases showed a significantly higher expression level than conventional non-recurrent ABs [54]. In the current study, most cases of AB and AC showed no expression of GPC-3. Of the cases of AB and AC that showed some degree of expression, no statistically significant findings were noted differentiating the two entities. Additionally, no differences were seen in the expression pattern between the cohort of ABs compared to ABs that ultimately transformed into ACs. Cases in the current study that showed expression of GPC-3 showed similar staining patterns as reported by Mendes et al*.*[13], with expression seen in the peripheral columnar cells. This expression pattern likely represents the stem-cell niche in the tumour, although further research is required to substantiate these findings. Given the molecular pathogenesis of these tumours, future studies comparing the molecular underpinnings of ABs and ACs are required to discover potentially novel molecular markers, followed by validation at the proteomic level with IHC, to better distinguish these entities.

In conclusion, the rarity of ACs and their challenging diagnosis, supports the notion that the histopathologic features should be correlated with clinical presentation and biological behaviour in reaching a definitive diagnosis. Further research is required to develop more stringent diagnostic histopathologic criteria to aid in diagnosing these rare entities. The current study found that using Ki-67 IHC as a proliferation marker, particularly when assessed via automated methods, was helpful in distinguishing AC from AB cases. Finally, in this study, surrogate IHC markers of embryonic stem cells, SOX2, OCT4 and GPC-3, were found to be unreliable in distinguishing AB from AC.

## Data Availability

The data of the current study is summarised in the tables and figures. Access to raw data is subject to approval by the University of Pretoria, Faculty of Health Sciences Research Ethics Committee.

## Code Availability

No software application or custom code was used in the preparation of this article.
